# A Practical Peptide Synthesis Workflow Using Amino-Li-Resin

**DOI:** 10.3390/mps5050072

**Published:** 2022-09-20

**Authors:** Damilola Caleb Akintayo, Srinivasa Rao Manne, Beatriz G. de la Torre, Yongfu Li, Fernando Albericio

**Affiliations:** 1 Peptide Science Laboratory, School of Chemistry and Physics, University of KwaZulu-Natal, Durban 4000, South Africa; 2 KwaZulu-Natal Research Innovation and Sequencing Platform (KRISP), School of Laboratory Medicine and Medical Sciences, College of Health Sciences, University of KwaZulu-Natal, Durban 4041, South Africa; 3 Biotide Core, LLC, 33815 SE Eastgate Circle, Corvallis, OR 97333, USA; 4 Institute for Advanced Chemistry of Catalonia (IQAC-CSIC), 08034 Barcelona, Spain; 5 CIBER-BBN, Networking Centre on Bioengineering, Biomaterials and Nanomedicine, Department of Organic Chemistry, University of Barcelona, 08028 Barcelona, Spain

**Keywords:** amino-Li-resin, solid-phase peptide synthesis, shaking, gravity filtration

## Abstract

Herein we report a practical approach for peptide synthesis using second-generation fibrous polyacrylamide resin (Li-resin, “Li” is coming from the name of its inventor, Yongfu Li). This resin with the corresponding handle was used for solid phase peptide synthesis (SPPS) using a fluorenylmethoxycarbonyl (Fmoc) approach. We reveal that the most appropriate mixing and filtration strategy when using amino-Li-resin in SPPS is via shaking and gravity filtration, instead of mechanical stirring and suction filtration used with other resins. The strategy was demonstrated with the SPPS of H-Tyr-Ile-Ile-Phe-Leu-NH_2_, which contains the difficult sequence Ile-Ile. The peptide was obtained with excellent purity and yield. We are confident that this strategy will be rapidly implemented by other peptide laboratories.

## 1. Introduction

Peptides are a key class of organic compounds. They are chemically synthesized by assembling two or more amino acids, which are joined by an amide bond formed from the interaction of the carboxylic end of one amino acid with the amino end of another [1,2,3,4]. Various classes of peptides have been reported to have multifarious pharmaceutical applications [5,6,7] for the treatment of a wide range of conditions, including cancer [8,9,10], metabolic syndrome [11,12,13], and infectious diseases [14,15,16,17]. Furthermore, peptides are constituent parts of vaccines [18,19,20] and are also used in cosmetics [21,22,23] and functional food [24,25].

Given the multidimensional use of peptides, efforts have been made over the years to optimize synthetic strategies for their preparation [1,26,27,28,29,30,31,32,33]. In this regard, four prominent approaches are used, namely classical solution, solid phase (on solid support) (SPPS) [34,35,36,37], hybrid [38] (which is a combination of classical solution and SPPS—in which protected peptides are prepared using SPPS and then are combined in solution), and liquid-phase peptide synthesis (LPPS) [39] (which follows the roots of SPPS and in which the growing chain is bound to a soluble tag). While the solution approach is laudable for its scalability in industrial production, it is limited with respect to the synthesis of longer peptide sequences, which are used mostly for therapeutic applications. Thus, as classical solution synthesis is tedious and time-consuming, in practice it is used only for the preparation of small peptides of up to 12 residues. In contrast, SPPS, which shows overall efficiency in terms of final purity and time and labor required, is currently the strategy of choice for the preparation of peptides for both research and industrial purposes. Furthermore, it is continuously being tweaked through various optimization strategies: the development of new resins [35,36,40,41,42,43], coupling agents [44], and protecting groups [45,46], and the use of green and benign solvents [29,30], and other strategies [26]. The hybrid approach, which combines classical solution and SPPS, has been demonstrated to be effective for the large-scale synthesis of longer peptides [47]. LPPS, which is being rapidly adopted by the industrial sector for the production of peptides, has the advantages of the classical solution strategy (in which reactions are performed in solution) and SPPS as the process is facilitated because the growing peptide chain is anchored to a soluble tag, which differentiates the peptides from the reagent used for elongation, thereby enhancing the purification process [48].

The most critical aspect of SPPS is the resin, which must have particular features to be suitable for peptide synthesis. In this regard, the resin should have an enhanced capacity to hold the different reactive sites (loading > 0.5 mmol/g), which should be uniformly distributed to minimize interchain interactions and therefore facilitate elongation of the peptide chain. It should also have good swelling properties in the solvents used to carry out the different reactions, thus enabling access of the reagents to the reactive sites. Moreover, the resin should be mechanically stable and permit filtration for removing the excess of reagents and soluble side products.

Polystyrene (PS)-based resins cross-linked with 1% of divinylbenzene are most commonly used in research laboratories and in large-scale peptide production by SPPS. Given that dichloromethane (DCM), which has been reported as hazardous, is the solvent of choice for swelling, the practicability of these resins is compromised. Similarly, PS-based solid support is further limited by its poor swelling capacity, particularly in polar solvents, which are the most adaptive for the synthesis of large peptides. In an attempt to change the paradigm (PS and DCM), significant efforts have been made to modify existing resins by increasing crosslinking or by adding new functionalities, thereby leading to new supports such as polyethylene glycol-PS (PEG-PS) [35,40,49,50] and cross-linked-ethoxylate-acrylate-resin (CLEAR) [51] (Table 1). Furthermore, PEG-based (ChemMatrix) [36,52] and polyacrylamide-based (SPAR-50) [53] resins have similarly gained much attention (Table 1). However, these resins have several drawbacks, such as low loading capacity, acid lability, either poor or over-swelling, difficulty to manufacture, and high cost.

To overcome the poor swelling capacity of PS and uneven solvation of PEG in some solvents, which is associated with major resins in the market today, there is a need to find a solid-phase support that is more homogeneous in terms of swellability and more versatile in terms of compatibility with the reaction conditions commonly used for solid-phase synthesis. Recently, our group described the second generation of amino-polyacrylamide resin (amino-Li-resin, “Li” is coming from the name of its inventor, Yongfu Li) for SPPS [37]. This support shows good swelling capacity in most polar organic solvents, including the green solvents most commonly used in SPPS.

An additional advantage of amino-Li-resin compared to PS and other resins is its excellent swelling capacity in H_2_O and even in aqueous buffers. Moreover, amino-Li-resin has a high loading capacity and good chemical stability towards acidic and basic reagents. Here we describe a practical protocol for the use of the amino-Li-resin for SPPS using the synthesis of Ile^2^,Ile^3^-Leu-Enkephalin (H-YIIFL-NH_2_) as a model.

## 2. Experimental Section

### 2.1. Materials and Reagents

All procedures pertaining to the peptide synthesis were carried out in a polypropylene syringe fitted with a penetrable frit. The HPLC grade solvents used in this study, namely dichloromethane (DCM), *N,N’*-dimethylformamide (DMF), acetonitrile (CH_3_CN), and methanol (MeOH), were purchased from Merck Pty, SA and used without further purification. The second-generation polyacrylamide resin (amino-Li-resin) with a 100–200 mesh particle size used herein was obtained from Biotide Core, LLC, Corvallis, OR, USA, and Fluorenylmethoxycarbonyl (Fmoc) amino acids were purchased from Iris Biotech GmbH (Marktredwitz, Germany). The coupling reagents *N,N’*-diisopropylcarbodiimide (DIC), OxymaPure^®^, and 1-[(1-(cyano-2-ethoxy-2-oxoethylideneaminooxy)-dimethylamino-morpholinomethylene)] methanaminium hexafluorophosphate (COMU^®^) were a gift from Luxembourg Biotech, Israel. The rest of the reagents, such as piperidine (deprotecting agent), *N,N*-diisopropylethylamine (DIEA), trifluoroacetic acid (TFA) (cleavage), and triisopropylsilane (TIS) (scavenger for the global deprotection and cleavage), were also supplied by Merck Pty Ltd. (Modderfontein, South Africa) and used in this protocol as received.

### 2.2. Instrumentation

All shakings were performed using a shaker operating at 120 rpm (Labcon, Gauteng, South Africa). Centrifugation was performed on a centrifuge (Apex Scientific, Durban, South Africa). Peptide purity was determined by means of an Agilent 1100 HPLC system using a Phenomenex AerisTMC18 (3.6 μm, 4.6 × 150 mm) column, with a flow rate of 1.0 mL/min and UV detection at 220 nm. 10 µL of the pentapeptide was injected on a reversed-phase C_18_ column (4.6 × 150 mm, 5 µm) operating at 1.0 mL/min, with linear gradients of 0.1% TFA in MilliQ water and 0.1% TFA in CH_3_CN as eluents. Data processing was performed using the Chemstation software (B.02.01 SR1 version) (Agilent, Santa Clara, CA, USA). Similarly, Liquid Chromatography-Mass Spectrometry (LCMS) analysis was conducted on an Ultimate™ 3000, Aeris^TM^ (Thermo Fisher Scientific, Waltham, MA, USA) with a Phenomenex C_18_ column (3.9 × 150 mm, 5 µm), a flow rate of 1.0 mL/min and UV detection of 220 nm, with a linear gradient of 0.1% formic acid in MilliQ water and 0.1% formic acid in CH_3_CN as eluents.

### 2.3. Synthesis Protocol

#### 2.3.1. Loading Determination

Amino-Li-resin (100 mg, ca. 0.08 mmol) was added to a 5 mL polypropylene syringe and subjected to swelling for 10 min in DMF (5 mL). The swelled resin was then pretreated with 5% DIEA in DCM (2 mL × 2) to neutralize the starting resin and then washed in DMF (2 mL × 2). A mixture of 5 eq. of Fmoc-Leu-OH (141.2 mg, 0.4 mmol), 5 eq. of DIC (62.5 µL, 0.4 mmol), and 5 eq. of OxymaPure (56.8 mg, 0.4 mmol) dissolved in DMF (1.5 mL, 0.2667 M) was added to the washed resin and shaken for 3 h. The loaded resin was washed with DMF (3 mL × 2) and MeOH (3 mL × 2) and left to dry until constant weight. Next, 20 mg of the loaded resin was weighed and transferred to a polypropylene syringe fitted with a porous frit, and 20% piperidine in DMF (500 µL) was added and stirred for 10 min. With the aid of a piston, the filtrate was gently pushed through into a volumetric flask (25 mL) and rinsed further with DMF (3 mL × 3). The solution was then made up to the mark with DMF.

Using a cuvette with a path length of 1 cm, the UV absorption profile for the solution was recorded using a UV spectrophotometer in the 270–320 nm range. The peak of the diagnostic dibenzofulvene adduct in the 298–301 nm range was used to determine the concentration of solution according to Equation (1), using a molar absorption coefficient ε of 7.33 L mmol^−1^ cm^−1^, obtained from the calibration curve of a freshly prepared Fmoc-Leu-OH solution. Using Equations (2) and (3), resin loading was then determined to be 0.4 mmol/g.

(1) $$c=\frac{A}{\varepsilon \times l}$$

(2) n=c×v

(3) $$L=\frac{n}{m}$$

*L* = Resin loading; *c* = concentration; *v* = volume of solution (volumetric flask); ε = molar absorption coefficient; *A* = absorption maxima around 298–301 nm, *l* = path length of the cuvette.

#### 2.3.2. Resin Swelling

**Figure 1:**
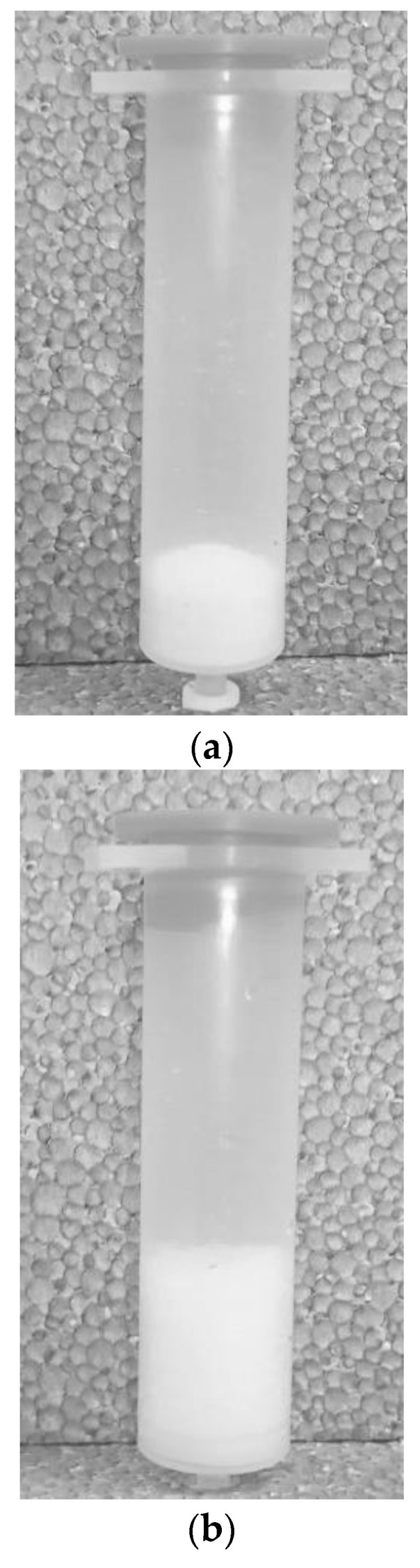
Amino-Li-resin weighed (**a**) Before swelling (**b**) After swelling.

Amino-Li-resin (0.4 mmol/g, 500 mg, 0.2 mmol scale) was added to a 20 mL polypropylene syringe fitted with a porous frit of 0.45 µm (Figure 1a). Next, 10 mL of DMF solvent was added to the resin and left for about 15 min to ensure resin swelling to maximum volume, after which the solvent was carefully removed by gravity filtration. The resin swelled to about twice its original volume (Figure 1b).

#### 2.3.3. Mixing and Filtration Strategy

**Figure 2:**
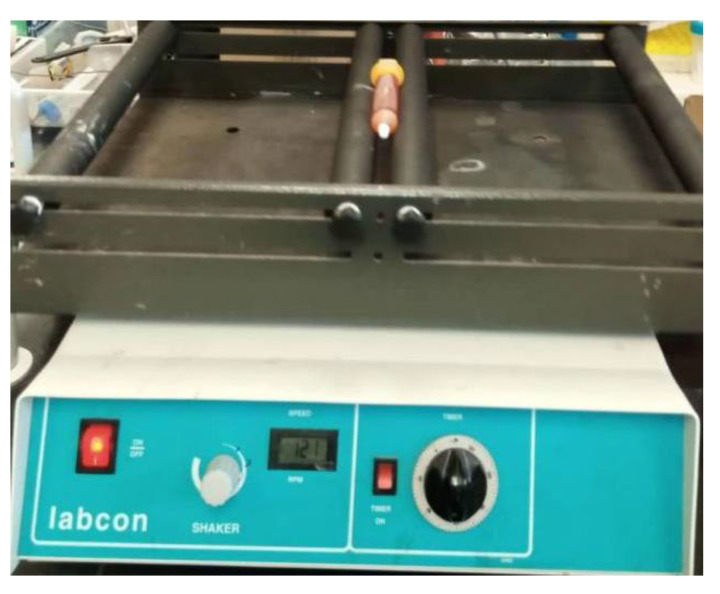
Shaking during the reaction of Fmoc Rink-Amide linker/COMU/DIEA.

In SPPS, resins are conventionally mixed by mechanical stirring, and filtration is performed using a vacuum source, both procedures tending to collapse the resin and clog the porous frit, thereby hampering the effectiveness of the entire process. To circumvent this deterioration, amino-Li-resin was gently mixed with the reagents by shaking (Figure 2) and filtration was performed by gravity, that is, the removal of excess reagent and washing steps were allowed to drain under gravity. 

**Figure 3:**
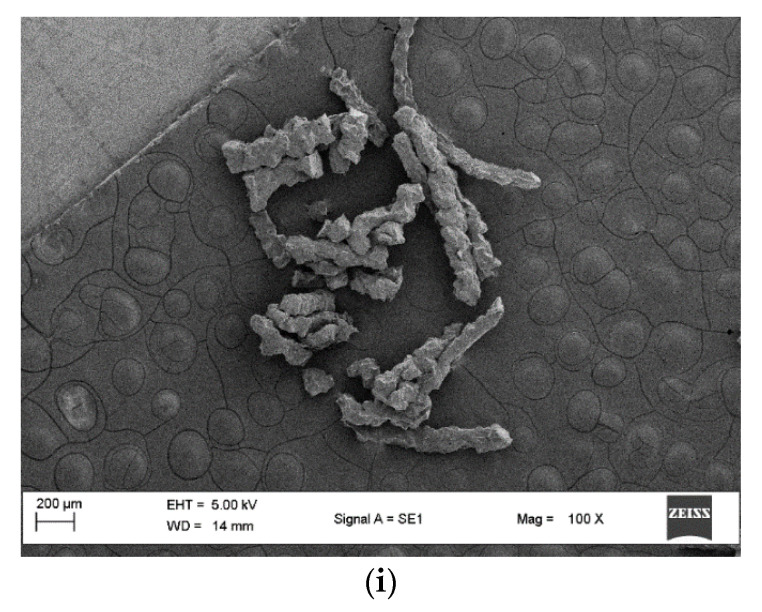

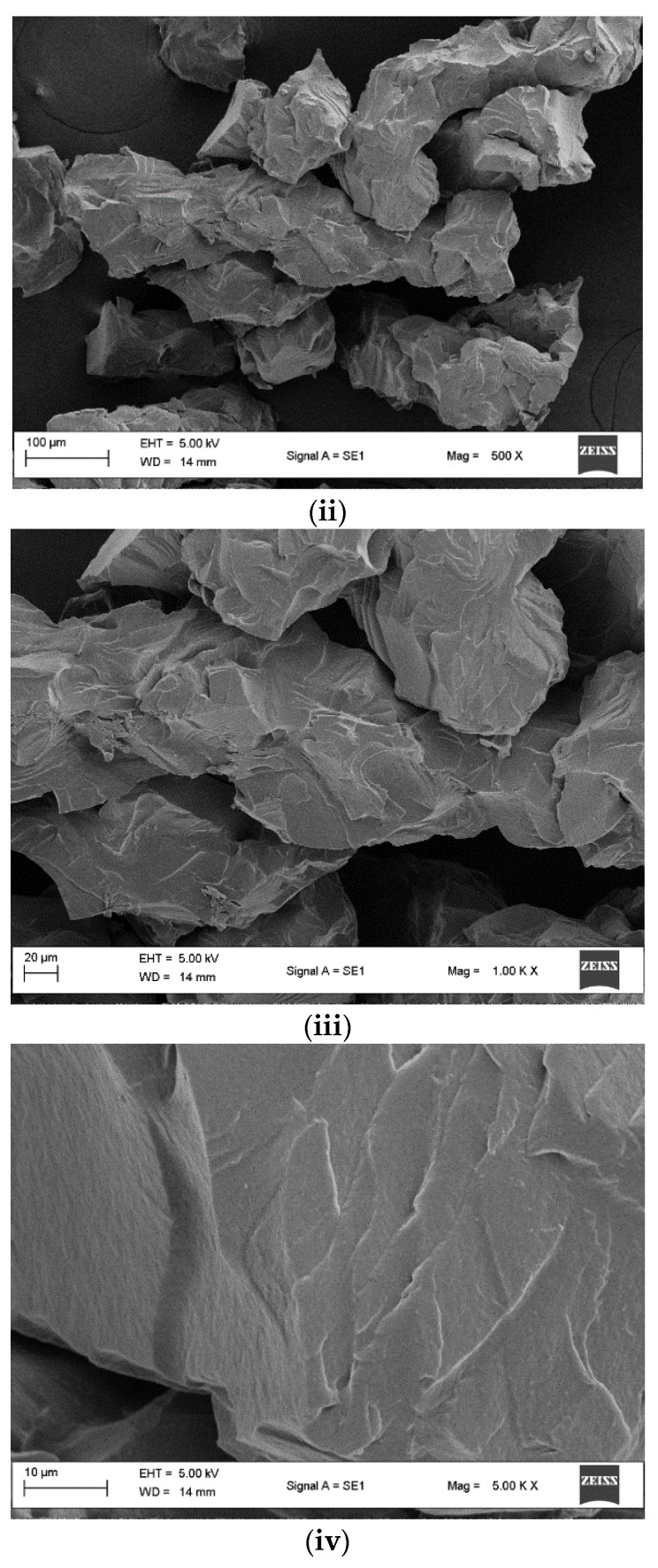
SEM images of amino-Li-resins after SPPS using shaking and gravity filtration: (**i**) 200 µm; (**ii**) 100 µm; (**iii**) 20 µm; and (**iv**) 10 µm.

**Figure 4:**
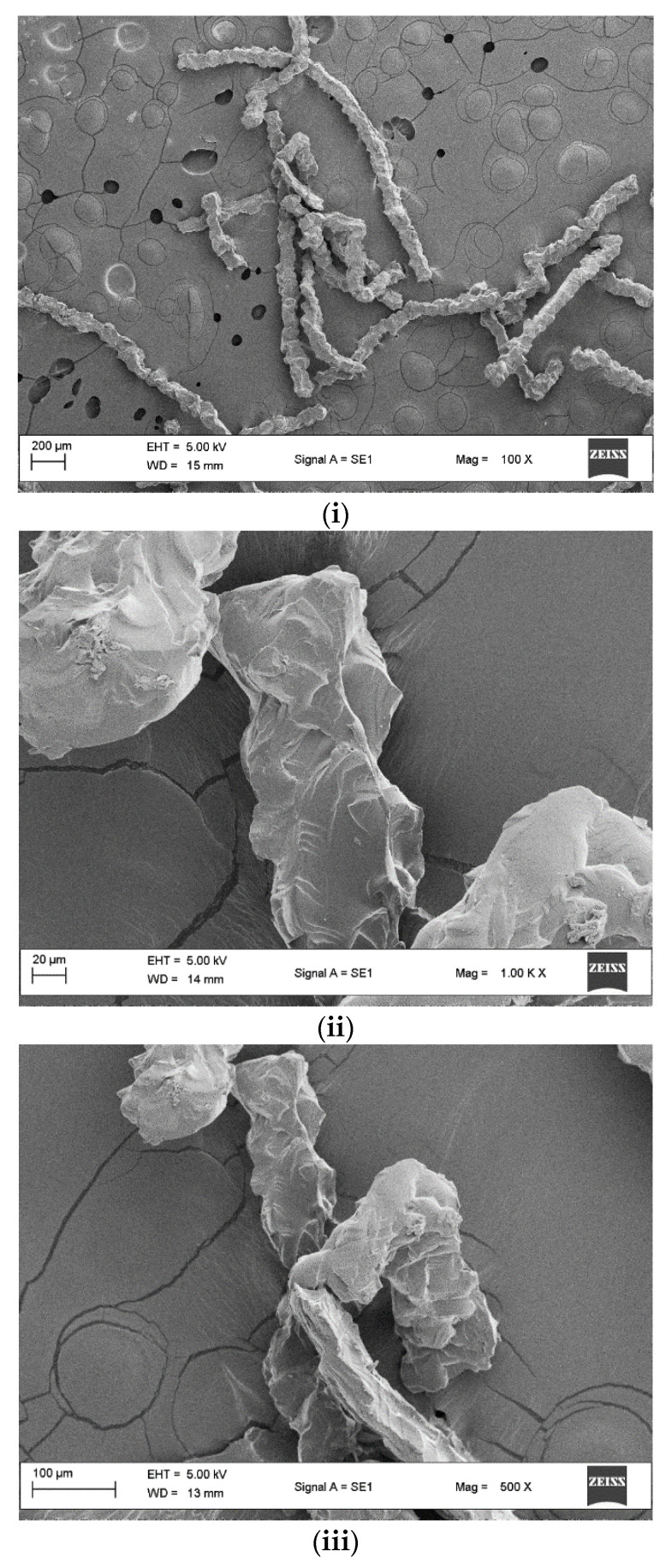

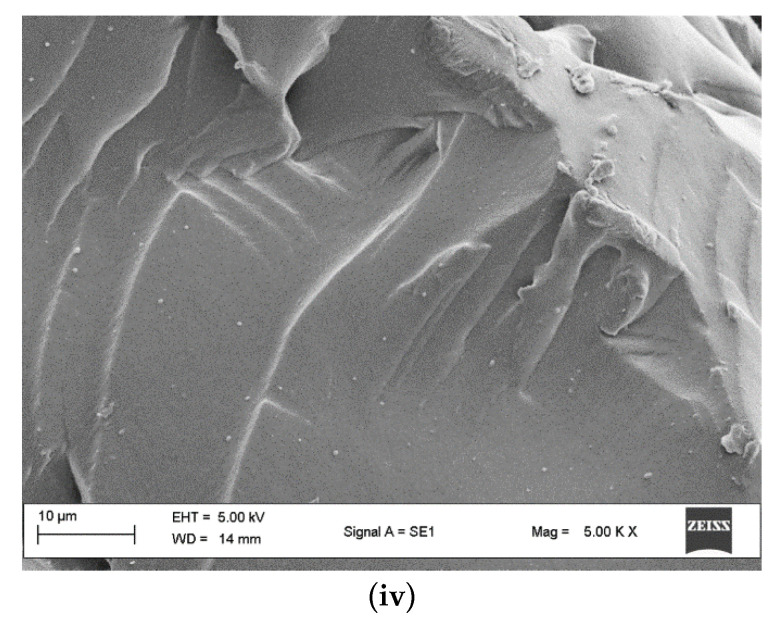
SEM images of crude amino-Li-resins: (**i**) 200 µm; (**ii**) 100 µm; (**iii**) 20 µm; and (**iv**) 10 µm.

After completion of the SPPS, images of the resin (Figure 3) using a scanning electron microscope (SEM) were compared to those of the resin before the synthesis (Figure 4). The results revealed that our methodology ensures the integrity of the resin.

#### 2.3.4. Addition of Fmoc Rink-Amide Linker

**Scheme 1:**
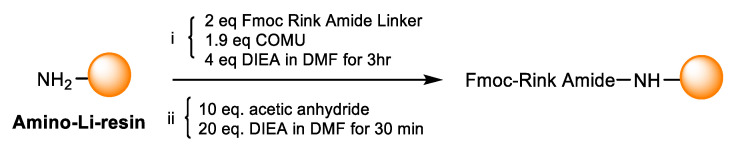
Anchoring of Fmoc Rink-Amide linker.

Fmoc Rink-Amide linker was incorporated into the amino-Li-resin by reacting the swelled resin with 4 eq. of Fmoc Rink-Amide linker (431.7 mg, 0.8 mmol), 3.8 eq. COMU (325.5 mg, 0.76 mmol), a slightly lower amount of COMU was used to prevent the capping of the amino function with the formation of the corresponding guanidino derivative [31] and 8 eq. DIEA (278.2 µL, 1.6 mmol) in DMF (3 mL) for 3 h (Scheme 1, Figure 4). The Fmoc Rink-Amide-Li-resin was then filtered by gravity, washed with DMF (5 mL × 3), filtered again by gravity, and then capped with the addition of 10 eq. acetic anhydride (Ac_2_O) (377.8 µL, 2 mmol) and 20 eq. of DIEA (1390.8 µL. 4 mmol) in DMF (3 mL) for 30 min. The solvent was then drained by gravity filtration and the resin was washed with DMF (5 mL × 3). Similar to what was established in Section 2.3.1 above, the loading of the Fmoc Rink-Amide linker onto the amino-Li-resin was assessed in triplicates and averaged to be 0.4 mmol/g.

#### 2.3.5. Fmoc-AA-OH Coupling

##### Deprotection

**Scheme 2:**
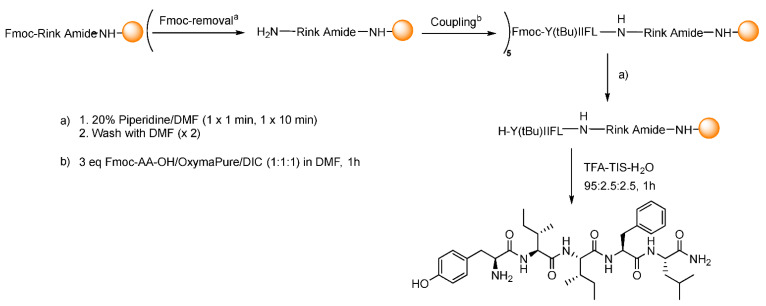
Schematic illustration of the synthesis of H-YIIFL-NH_2._

Elongation of the sequence proceeded in a continuous cycle (Scheme 2) beginning with the removal of the 9-fluorenylmethyloxycarbonyl (Fmoc) group on the Fmoc Rink-Amide-Li-resin using 20% piperidine in DMF (5 mL). This deprotection step was performed at RT for 10 min, and the solution was then drained under gravity and washed with DMF (5 mL × 2).

##### Coupling

The first amino acid was incorporated by the addition of a preactivated mixture (0.3 M) of 3 eq. Fmoc-Leu-OH (423.6 mg, 0.6 mmol) containing 3 eq. OxymaPure (170.4 mg, 0.6 mmol) and 3 eq. DIC (187.6 µL, 0.6 mmol) to the deprotected Rink-Amide-Li-resin, followed by gentle shaking for 1 h (Figure 4). The solvent was removed by gravity and the peptidyl resin was subjected to deprotection by the addition of 20% piperidine in DMF (5 mL). The incorporation of other Fmoc-AA-OH was achieved in a like manner.

##### Cleavage

After the final deprotection step in the last incorporation, the peptidyl resin was subjected to a final cleavage to detach the peptide. The cleavage cocktail solution was a freshly prepared mixture of TFA (12.35 mL), TIS (0.325 mL), and water (0.325 mL) in 95%:2.5%:2.5% ratio. The cocktail (1 mL per 100 mg of peptidyl resin) was added to a falcon tube containing the peptidyl resin and left to stir for 1 h. Given that TFA is highly corrosive, it should be used in a well-ventilated environment and discarded in an approved waste disposal plant.

##### Precipitation

The peptide was precipitated after cleavage by the addition of 45–70 mL of cold diethyl ether (usually 5–8 times the cleavage solution) followed by intensive agitation, leading to the appearance of a whitish precipitate. The falcon tube was then centrifuged (3600 rev per min for 4 min). The supernatant layer was carefully decanted into a clean falcon tube and kept in ice for further precipitation of peptides that might have been released during decantation. The cleaved peptide was then washed twice with cold diethyl ether in the same manner and dried in vacuo to completely remove the ether. The peptide was finally extracted with 10% acetic acid (HOAc), then filtered under gravity, frozen with liquid nitrogen, and kept in a freeze dryer for 24–48 h until a powder was obtained.

## 3. Result

### Yield and Purity

The Ile^2^,Ile^3^-Leu-Enkephalin (H-YIIFL-NH_2_) peptide after lyophilization ($W_{P}$) had a dry weight of 108.03 mg, as determined on a mass balance. The theoretical yield ($Y_{theor}$) of the peptide was determined from Equations (4) and (5). Given that 0.5 g of a 0.4 mmol/g of amino-Li-Resin was used in this study, according to Equation (4), the reaction scale (*R_S_*) was 0.2 mmol. Furthermore, according to Equation (5), the theoretical yield ($Y_{theor}$) was 133.37 mg. Hence, according to Equation (6), the peptide yield ($Y_{Peptide}$) was 81% (not optimized).

(4) $$R_{S}=M_{R}\times R_{L}$$

(5) $$Y_{theor}=R_{S}\times M_{W}\;\mathrm{of}\;\mathrm{the}\;\mathrm{peptide}$$

(6) $$Y_{Peptide}\;=\;(\frac{W_{P}}{Y_{theor}})\;\times \;\mathrm{100}$$

$R_{S}$ = Scale of reaction, $R_{L}$ = Resin loading, $M_{R}$ = Mass of resin used, *M_w_* = Molecular weight of the peptide, *W_p_* = Dry weight of peptide after lyophilization.

**Figure 5:**
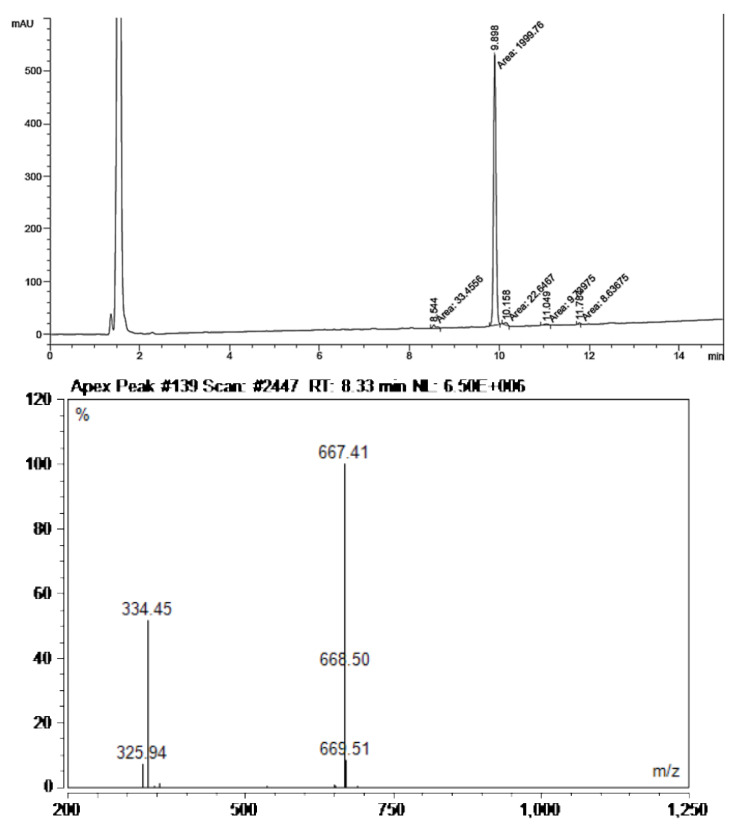
(**Top**) HPLC chromatogram; (**Bottom**) LCMS spectra of H-YIIFL-NH_2_ after precipitation.

As shown in the chromatogram (Figure 5) obtained from the HPLC analysis, the model pentapeptide (H-YIIFL-NH_2_) had purity of 96.1%, thus confirming the suitability of the amino-Li-resin for SPPS.

## 4. Conclusions

Amino-Li-resin is a second-generation polyacrylamide support with a highly cross-linked matrix designed to confer the mechanical stability required for effective use in SPPS. It has excellent swelling capacity in a wide range of solvents, particularly in polar solvents such as water and alcohols, which are compatible with biochemical processes. The suitability of amino-Li-resin has been demonstrated in a stepwise synthesis of Ile^3^, Ile^4^ -Leu Enkephalin using the Fmoc/tBu strategy via shaking and gravity filtration, which assures the integrity of this resin. Unlike the ChemMatrix resin, which has a very low yield, amino-Li-resin afforded a relatively high yield (81%) in the synthesis of the model peptide used in this protocol.

## Figures and Tables

**Table 1:** Common resins used in SPPS and their chemical structure.

S/N	Chemical Structure	Name	Ref
1	Cross-linked Polystyrene	Polystyrene	[34]
2	Polyethylene glycol-polystyrene (PEG-PS) graft	PEG-PS, TentaGel, Octagel, HiCore	[35,40,49,50]
3	Crosslinked Polyethylene glycol	ChemMatrix	[36,52]
4	Crosslinked Polyacrylate PEG	CLEAR	[51]
5	Polyacrylamide	Amino-Li-resin, SPAR50	[37,53]

## Data Availability

The data presented in this study are available within the article.
